# Iatrogenic Gastrointestinal Injuries During Obstetrical and Gynecological Operation

**DOI:** 10.5812/atr.12088

**Published:** 2013-08-01

**Authors:** Elaheh Mesdaghinia, Masoumeh Abedzadeh-Kalahroudi, Mehrdad Hedayati, Nushin Moussavi-Bioki

**Affiliations:** 1 Department of Obstetrics and Gynecology, Kashan University of Medical Sciences, Kashan, IR Iran; 2 Trauma Research Center, Kashan University of Medical Sciences, Kashan, IR Iran; 3 Deputy of Health, Kashan University of Medical Sciences, Kashan, IR Iran; 4 Department of General Surgery, Kashan University of Medical Sciences, Kashan, IR Iran

**Keywords:** Injuries, Gynecology, Surgery

## Abstract

**Background:**

Gastrointestinal Injuries (GI) during gynecological operation are uncommon but proper management of these injuries is very important.

**Objectives:**

The aim of this study was to review the causes and management of gastrointestinal injuries during gynecological and obstetrical operations.

**Patients and Methods:**

In this descriptive retrospective study, 25 patients with gastrointestinal injuries during gynecological and obstetrical operation at Shabihkhani Maternity Hospital in Kashan city were reviewed. Demographic data such as age, gravid, parity, type of surgery or procedure, history of laparotomy, the surgical operation, injury site, time of diagnosis and method of treatment were extracted from medical records.

**Results:**

The mean age of women was 33.2 ± 7.57 years. Fourty-four percent of the patients had a history of abdominal scar. Thirty-two percent of all GI injuries occurred during total abdominal hysterectomy (TAH). The small bowel was injured in 36% of cases. Fifty-two percent of injuries were diagnosed during the operation and the mean time of injury diagnosis was 2.8 ± 0.9 days.

**Conclusions:**

All of the gynecologic surgeons must be aware of gastrointestinal injuries and should anticipate injury to these organs especially in high-risk patients for decreasing patient morbidity.

## 1. Background

Gastrointestinal Injuries are one of the complications during gynecological operation (1). Small intestine and colon injuries can be seen during gynecological procedures from dilatation and curettage (D&C) to total abdominal hysterectomy (TAH) and laparoscopic or hysteroscopic procedures. Colon injuries can occur in patients with left adnexal mass and women with a history of pelvic inflammatory disease or diverticulitis (2). The common places of injury are the large bowel, small bowel, rectum and rarely the gastric region (3, 4). The incidence of bowel injuries is different based on the type of operation, ranging from 0.62 - 1.6 per 1000 laparoscopic surgeries (5), to 0.3% in hysterectomy (6), 0.08% in cesarean section (C/S) (7) and 0.04% in normal vaginal delivery (NV/D) (8). Also, overall incidence of bowel injuries is between 0.54% - 0.7% (9, 10). Approximately one-third of bowel injuries may be diagnosed during the operation (5, 9, 10). Mild injuries to the small and large bowel can be repaired by intestinal closure but extensive injuries need a resection (11).

Based on our knowledge, there are a few published researches on the incidence of gastrointestinal injuries during gynecologic operation. In one study on 128 bowel injuries, the rate of injuries during "opening of the peritoneal cavity", "adhenolysis and pelvic dissection", "laparoscopy", "vaginal operations" and "dilatation and curettage" was 37.5%, 35.2%, 10.2%, 8.6%, and 8.6%, respectively. Seventy-five percent of all injuries were to the small bowel and 25% were to the large bowel (10). Also, in another study, the rate of bowel injuries was similar to previous research and in this research all injuries were repaired by intestinal closure, resection of small bowel or colostomy (9). Proper management of injuries during obstetric and gynecologic operation is very important (11) and all gynecologists must be aware of such injuries to decrease patient morbidity and prevent mortality (12).

## 2. Objectives

Considering the importance of this issue and lack of published data in Iran, this study was performed to determine the causes and management of iatrogenic gastrointestinal injuries during gynecological and obstetrical operation over a period of 12 years in Shabihkhani Maternity Hospital in Kashan, Iran.

## 3. Materials and Methods

In this descriptive retrospective study, performed from 1999 - 2011, all iatrogenic gastrointestinal traumas (n=25) during gynecological and obstetrical operations in Shabihkhani Maternity Hospital in Kashan city were studied. Shabihkhani Maternity Hospital is an educational hospital for training of obstetric and gynecologic residents. Demographic data such as age, gravid, parity, type of surgery or procedure, history of laparotomy, the surgical operation, injury site, time of diagnosis and method of treatment were extracted from the medical records. Data were analyzed using descriptive statistics.

## 4. Results

Over a period of 12 years, there were 25 cases of gastrointestinal injuries during gynecological and obstetrical operations. The mean age of women was 33.2 ± 7.57 years, the mean number of gravid was 2.44 ± 1.2 and the mean number of parity was 1.8 ± 1.2. In total 44% of patient had a history of cesarean section and 64% of them had a history of vaginal delivery. Table 1 shows that 44% of patients had a history of abdominal scar. In 36% of cases the cause of injury was the fault of a resident of obstetrics and gynecology. Thirty-two percent of all gastrointestinal injuries occurred during total abdominal hysterectomy. The small bowel was injured in 36% of the cases. 

**Table 1.** Distribution of the Frequency and Valid Percentage of Some Variables in Patients With GI Injuries

Variables	No.	Percent
**Type of operation**		
TAH ^a^	8	32
D&C ^a^	5	20
C/S ^a^	3	12
NV/D ^a^	3	12
Laparoscopy	2	8
Cystectomy	2	8
Culdocentesis	1	4
TL ^a^	1	4
**Injured organ**		
Small bowel	9	36
Cecum	6	24
Colon	5	20
Rectum and anus	4	16
Stomach	1	4
**Type of management**		
Primary repair	14	56
Colostomy and repair	10	40
No treatment	1	4
**Time of diagnosis**		
During operation	13	52
After operation	12	48
**Previous pelvic or abdominal surgery**		
Yes	11	44
No	14	56
**Surgeon**		
Resident	9	36
Specialist	11	44
Both	5	20

^a^ Abbriviations: C/S, cesarean section; D&C, dilatation and curettage; NV/D, normal vaginal delivery; TAH, total abdominal hysterectomy.

Regarding the type of surgery and injured organ, results showed that most of intestine injuries occurred during dilatation and curettage (44%). Also, most of the colon injuries occurred during cesarean section and total abdominal hysterectomy (33%). Moreover, 100% of cecum injuries were in patients who underwent total abdominal hysterectomy. Stomach injury was seen only in one case who had undergone ovarian cystectomy. In onethird of the surgeries, patients underwent laparotomy for peritonitis and stomach perforations were diagnosed and repaired. In one patient the cause of injury to the intestine was culdocentesis. During the operation, green discharge from the needle was observed that indicated insertion to the intestine but there was no need to repair. This patient was followed for one day and discharged from the hospital without any problems. Rectum injury occurred during vaginal delivery in three patients. In two patients who underwent laparoscopic surgery, small bowel and colon injury had similar rates (50%). In one case, laparoscopy was done for adhenolysis and the patient had a history of ovarian cystectomy and total abdominal hysterectomy. After diagnosis of colon injury, repair and colostomy was performed.

Fifty-two percent of the injuries were diagnosed during the operation and the mean time of injury diagnosis was 2.8 ± 0.9 days (Ranging from 0 to 4 days). In one patient 10 days after colostomy for cecum injury, recto vaginal fistula was found and repaired. Diagnosis of injury during the operation was based on clinical findings and diagnosis of injury after the operation was based on clinical symptoms such as pain, fever, ileus and diagnostic tests such as ultrasonography, X-ray CT scan and laparotomy.

Injury treatment for 56% of patients was primary repair (Table 1). Repair of 44% of all injuries was performed by a general surgeon. 

## 5. Discussion

Gastrointestinal injury incidental to obstetrical and gynecological operation is a rare event and lack of published information makes it harder to compare and review the findings. Our findings showed that 32% of all gastro intestinal injuries occurred in total abdominal hysterectomy. The most common site that was injured in total abdominal hysterectomy was the cecum (62.5%). Injuries during dilatation and curettage occurred in 20% of the cases. The most common injury site in dilatation and curettage was the small bowel (80%). There was only one gastric injury (4%) in this series that was seen in patients who had surgery for ovarian cysts. Usually gastric injury can be caused during laparoscopy (13). The small bowel was injured in 36% of cases. However in the study by kerb et al. (10), this rate was 75% while Bhatte et al found this rate to be 61.9% (9, 10); these results are different from our findings and this may be due to the type of surgery that involves mainly the large intestine. Fourty-four percent of the patients had a history of previous pelvic or abdominal surgery. In one study this rate was 64.3% (9). Patients with a history of abdominal surgery scar are at high risk for intestinal injuries (13). This may be due to intestinal adhesions following previous pelvic or abdominal surgery (11). Suspicion of high-risk patients, exact examination of all bowel and even intraoperative colonoscopy is recommended to prevent injuries and for early recognition and management (14). In 36% of cases, the leading cause of injury was due to the surgical errors of obstetrics and gynecology residents. In other studies, the surgical procedure was not mentioned and therefore it is not possible to compare the findings in this field. However, all of the obstetrics and gynecology residents must be trained in prevention and management of these injuries (9). Fifty-two percent of the injuries were diagnosed during the operation and 48% were diagnosed after the surgery. In a study on laparoscopic surgery, one-third of intestinal injuries were diagnosed during the operation (5). This difference may be because we reviewed all types of surgery but in the Sabharwal study, only injuries during laparoscopic hysterectomy were assessed. Missed bowel injuries are more common in laparoscopic surgery, where thermal injury to the bowel may not be obvious intra-operatively (15). In our study, 48% of injuries were diagnosed in the postoperative period. Considering that only 8 percent of the procedures were laparoscopic, this high percent of missed injuries is of concern. The mean diagnosis time of the injury was 2.8 ± 0.9 days (0 - 4 days). It was different from the Chapron et al study on laparoscopic operation, in which the mean diagnosis time of the injury was 4.0 ± 5.4 days (16).

Intra-operative detection of injuries is of great importance, since the delayed diagnosis increases morbidity, and repair of the injury in an infected and inflamed abdomen may even lead to placement of a colostomy (17). Management of injury in 56% of patients was primarily by repair. In one study all injuries were repaired by intestinal closure, resection of the small bowel or colostomy (9). Mild injuries to the small and large bowel can be repaired by intestinal closure but extensive injuries need resections (11). Today, all of the gynecologic surgeons must be trained in techniques to avoid and repair gastro intestinal injuries. Especially, when there is a previous abdominal scar, they should be cautious to enter peritoneal or pelvic cavity. As intra-operative colonoscopy is not available in many centers, the only way to decrease iatrogenic injuries and prevent missed injuries is the exact examination of the abdomen, gentle dissection of the tissues and high suspicion of high- risk patients with previous abdominal operations. Laparoscopic procedures are becoming more and more prevalent and special attention should be given to teach precise techniques and instrument utilization to prevent occult iatrogenic injuries.

## References

[1] Richter R (1981). [Prophylaxis and therapy of intestinal complications in surgical
gynecology].. Ther Umsch..

[2] Paloyan D, Tommaso F, William W, Paloyan D, Tommaso F, William W (2008). Clinical Reproductive: Medicine and Surgery.. Intestinal Problems in Gynecologic Surgery..

[3] Gonzalez RP, Merlotti GJ, Holevar MR (1996). Colostomy in penetrating colon injury: is it necessary?. J Trauma..

[4] Mendez LE (2001). Iatrogenic injuries in gynecologic cancer surgery.. Surg Clin North Am..

[5] Sabharwal M (2009). Large bowel injury during total laparoscopic hysterectomy.. J Gynecol Endosc Surg..

[6] Kafy S, Huang JY, Al-Sunaidi M, Wiener D, Tulandi T (2006). Audit of morbidity and mortality rates of 1792
hysterectomies.. J Minim Invasive Gynecol..

[7] Jones OH (1976). Cesarean section in present-day obstetrics. Presidential
address.. Am J Obstet Gynecol..

[8] Nielsen TF, Hokegard KH (1984). Cesarean section and intraoperative surgical complications.. Acta Obstet Gynecol Scand..

[9] Bhattee GA, Rahman J, Rahman MS (2006). Bowel injury in gynecologic operations: analysis of 110
cases.. Int Surg..

[10] Krebs HB (1986). Intestinal injury in gynecologic surgery: a ten-year
experience.. Am J Obstet Gynecol..

[11] Davis JD (1999). Management of injuries to the urinary and gastrointestinal tract during
cesarean section.. Obstet Gynecol Clin North Am..

[12] Stany MP, Farley JH (2008). Complications of gynecologic surgery.. Surg Clin North Am..

[13] Rock JA, Jones HW (2011). TeLinde's Operative Gynecology..

[14] Nezhat C, de Fazio A, Nicholson T (2005). Intraoperative sigmoidoscopy in gynecologic surgery.. J Minim Invasive Gynecol..

[15] Baggish MS (2008). Lessons in timely recognition of laparoscopy-related bowel
injury.. JFPonlinecom..

[16] Chapron C, Pierre F, Harchaoui Y, Lacroix S, Beguin S, Querleu D (1999). Gastrointestinal injuries during gynaecological
laparoscopy.. Hum Reprod..

[17] Sweeny KJ, Joyce M, Geraghty JAMES G (2002). Management of intraoperative bowel injuries.. CME J Gynecol Oncol..

